# Soybean bioactive peptide supplementation improves gut health and metabolism in broiler chickens

**DOI:** 10.1016/j.psj.2024.104727

**Published:** 2024-12-22

**Authors:** Han Peng, Xiaoyan Song, Jialei Chen, Xia Xiong, Li Yang, Chunlin Yu, Mohan Qiu, Zengrong Zhang, Chenming Hu, Shiliang Zhu, Bo Xia, Jiangxian Wang, Zhuxiang Xiong, Longhuan Du, Chaowu Yang

**Affiliations:** Animal Breeding and Genetics key Laboratory of Sichuan Province, Sichuan Animal Science Academy, Chengdu 610066, PR China

**Keywords:** Soybean bioactive peptide, Yellow-feathered broilers, Growth performance, Intestinal health, Metabolism

## Abstract

This study aimed to investigate the effects of soybean bioactive peptide (**SBP**) on the growth performance and intestinal health of yellow-feathered broilers and to further elucidate the regulatory mechanisms of intestinal health using multi-omics analysis. A total of 320 1-day-old yellow-feathered broilers were randomly divided into two groups, with 10 replicates per group and 16 birds per replicate. Broilers in the control group received the basal diet, and those in the experimental group (SBPG) received the basal diet with 0.2 % SBP replacing the same amount of soybean meal. The experiment lasted for 70 d. The results showed that, compared with those in the control group, the final body weight and average daily gain of SBPG broilers were significantly higher (*P* < 0.05), and the feed conversion ratio was significantly lower (*P* < 0.05). Notably, SBP significantly improved gut health in chickens, including increased intestinal villus height, decreased levels of proinflammatory factors, such as IL-1β and interferon-γ, and upregulated expression of tight junction proteins, such as *ZO-1* and *occludin.* In addition, transcriptome sequencing results revealed that broilers in the SBP group exhibited significant enrichment in multiple metabolic pathways, including fatty acid metabolism, fatty acid degradation, and the biosynthesis of unsaturated fatty acids (*P* < 0.05). Cecal 16S rRNA sequencing showed that SBPG increased the abundance of the butyrate-producing beneficial bacteria *Muribaculaceae*. Subsequent cecal metabolome analysis also revealed that SBPG enhanced lipid-related metabolic pathways, such as alpha-linolenic acid metabolism and GPI-anchor biosynthesis. In conclusion, SBP is a potential feed additive that can improve intestinal morphology, enhance intestinal immunity and barrier function, optimize the structure of the intestinal microbiota, and enhance metabolic function.

## Introduction

Yellow-feather broilers possess the advantages of strong stress resistance, high feed conversion ratio, and delicious meat, making them popular among individual and family consumers (Guo et al., 2023). Improving their production performance is a key focus for future development. The intestine is a key factor affecting the health and growth performance of poultry. Currently, various stressors present in intensive farming can easily cause intestinal damage in broilers, thereby reducing immunity and feed efficiency, which results in growth retardation, increased morbidity, and even broiler death (Caekebeke et al., 2020; Sugiharto, 2022). Therefore, maintaining intestinal health is critical to improving yellow-feathered broiler production. There is growing evidence that functional dietary peptides can improve the intestinal health of broilers, thereby enhancing intestinal digestive and absorptive functions and disease resistance, ultimately improving growth performance and overall health (Karimzadeh et al., 2016; Osho et al., 2019; Landy et al., 2020).

Soybean bioactive peptide (**SBP**), a promising dietary peptide, is a mixture of oligopeptides obtained by the hydrolysis or microbial fermentation of soybean protein. Compared to traditional soy protein, SBP exhibits stronger stability, better digestion and absorption, and multiple functional activities, such as antioxidant, anti-hypertensive, antidiabetic, and anticancer properties (Chatterjee et al., 2018). In addition, adding an appropriate amount of SBP to animal feed can improve intestinal morphological development, enhance immune and intestinal barrier functions, and regulate intestinal microbiota, thereby promoting the healthy growth of animals (Abdollahi et al., 2017; Wei et al., 2024). Moreover, SBP can effectively repair intestinal damage caused by pathogenic bacterial infections and support animal production (Zhang et al., 2019). Therefore, SBP is a high-value, multifunctional feed additive with broad application prospects in livestock and poultry production. However, the effects and potential mechanisms of SBP on the intestinal health of yellow-feathered broilers remain unclear. The possible mechanisms of SBP have been evaluated comprehensively from the perspectives of intestinal structure, intestinal flora, and metabolites.

In the present study, we investigated the effects of SBP on the growth performance and intestinal health of yellow-feathered broilers. We applied transcriptome, microbiome, and metabolome analyses to reveal the possible mechanisms by which SBP regulates intestinal health and promotes growth from three perspectives: gene regulation, intestinal microbiota, and metabolites. These findings provide a theoretical basis and technical support for SBP as a functional additive while facilitating the healthy and rapid development of the yellow-feathered broiler industry.

## Materials and methods

### Ethics statement

Animal experiments were conducted in accordance with the Regulations on the Administration of Affairs Concerning Experimental Animals (Ministry of Science and Technology, China). All animal experiments were approved by the Animal Ethics Committee of the Sichuan Animal Science Academy (approval no. 0235/2022).

### Animal experiment and sample collection

A total of 320 1-day-old healthy, medium-growing, yellow-feathered male broiler chickens were randomly assigned to control and SBP groups. Each group consisted of 10 replicates, with 16 birds per replicate. The chickens’ diets were formulated to meet or exceed the feeding standards for chickens in China (NY/T 33-2004) (Table 1). The diet of chickens in the SBP group was supplemented with 0.2 % SBP (Chengdu Mytech Biotech Co. Ltd., Chengdu, Sichuan, China; Table 1), replacing the same amount of soybean meal. Feed and water were provided ad libitum, and mortality was monitored daily. The experiment lasted 70 d. Body weight and feed intake were recorded on a per-replicate basis at the beginning and end of the experiment, and average daily gain, average daily feed intake, and feed conversion ratio were calculated. The feed conversion ratio was corrected for mortality.

**Table 1:** Composition and nutrient levels of the basal diet (air-dry basis).

Items	1 to 21 days of age	22 to 42 days of age	43 to 70 days of age
Ingredients, %			
Corn	61.70	66.40	69.49
Soybean meal	25.70	20.60	17.00
Corn gluten meal	4.00	4.00	4.00
Corn DDGS	3.00	3.00	3.00
Soybean oil	1.00	1.50	2.60
Limestone	1.30	1.40	1.30
CaHPO_4_	1.50	1.30	1.00
NaCl	0.26	0.26	0.26
*L*-Lysine	0.38	0.32	0.30
*DL*-Methionine	0.20	0.18	0.11
*L*-Threonine	0.06	0.08	0.07
Choline chloride	0.08	0.10	0.07
Premix	0.82 1)	0.86 2)	0.80 3)
SBP 4)	0.00	0.00	0.00
Total	100	100	100
Nutrient levels 5)			
ME, kcal/kg	2900	2970	3080
CP, %	19.57	17.68	16.30
Calcium, %	0.92	0.89	0.78
Total phosphorus, %	0.60	0.56	0.49
Non-phytate phosphorus, %	0.38	0.34	0.30
Digestible lysine, %	1.10	1.00	0.90
Digestible methionine, %	0.50	0.47	0.39

SBP, soybean bioactive peptide.

1) The premix provided the following per kg of diet: Vitamin A 9,900 IU, Vitamin D_3_ 1,800 IU, Vitamin E 38 IU, Vitamin K_3_ 3.15 mg, Vitamin B_1_ 2.70 mg, Vitamin B_2_ 6.75 mg, Vitamin B_6_ 4.50 mg, Vitamin B_12_ 31.50 μg, biotin 0.23 mg, pantothenic acid 15.75 mg, folic acid 1.10 mg, nicotinic acid 45 mg, Fe 150 mg, Cu 19 mg, Mn 132 mg, Zn 110 mg, I 0.41 mg, Se 0.49 mg.

2) The premix provided the following per kg of diet: Vitamin A 9,240 IU, Vitamin D_3_ 1,680 IU, Vitamin E 35.7 IU, Vitamin K_3_ 2.94 mg, Vitamin B_1_ 2.52 mg, Vitamin B_2_ 6.30 mg, Vitamin B_6_ 4.20 mg, Vitamin B_12_ 29.40 μg, biotin 0.21 mg, pantothenic acid 14.70 mg, folic acid 1.05 mg, nicotinic acid 42 mg, Fe 135 mg, Cu 15 mg, Mn 110 mg, Zn 114 mg, I 0.53 mg, Se 0.50 mg.

3) The premix provided the following per kg of diet: Vitamin A 8,800 IU, Vitamin D_3_ 1,600 IU, Vitamin E 34 IU, Vitamin K_3_ 2.80 mg, Vitamin B_1_ 2.40 mg, Vitamin B_2_ 6.0 mg, Vitamin B_6_ 4.0 mg, Vitamin B_12_ 28 μg, biotin 0.20 mg, pantothenic acid 14 mg, folic acid 1.0 mg, nicotinic acid 40 mg, Fe 135 mg, Cu 15 mg, Mn 110 mg, Zn 114 mg, I 0.53 mg, Se 0.50 mg.

4) SBP, provided by Chengdu Mytech Biotech Co., Ltd., has a crude protein content of at least 55 %, an acid-soluble protein content (i.e., peptide content) of at least 30 %, and peptides with a molecular weight of 150 to 1,000 u (2–6 peptides) accounting for more than 18 %. Among them, the content of the antioxidant-active peptides Glu-Tyr and Ala-Phe reached milligram levels.

5) Nutrient levels were calculated values.

At 70 d of age, 10 chickens per group (one chicken per replicate) were randomly selected for sample collection. Ileum tissues were fixed in 4 % paraformaldehyde. The ileum mucosa was scraped using a sterile scraper, and the content from the cecum was collected and frozen in liquid nitrogen. Both ileum mucosa and cecal content samples were transferred to –80°C and stored for examination.

### Intestinal morphology evaluation

Ileum tissues were fixed in 4 % paraformaldehyde, embedded in paraffin wax, and sliced. After the ileum sections were stained with hematoxylin and eosin, the thickness of the ileal muscle, villus height (**VH),** and crypt depth were evaluated under a light microscope (Olympus, Japan) equipped with an image analyzer (Image-Pro Plus 6.0). The distribution of goblet cells in the intestinal epithelium and the mucous layer thickness were observed using a light microscope equipped with an image analyzer (Image-Pro Plus 6.0) after staining the sections with Periodic Acid-Schiff stain.

### Intestinal immune factor analysis

Concentrations of IL-1β, IL-6, IL-10, interferon-γ (IFN**-γ**), tumor necrosis factor-α (TNF-α), and secretory immunoglobulin A (**SIgA**) in the ileum mucosa of chickens were evaluated using commercial chicken ELISA kits (ZCIBIO Technology Co., Ltd., Shanghai, China) according to the manufacturer's recommendations.

### Real-time quantitative PCR

Real-time quantitative PCR was used to detect mRNA levels of genes involved in the regulation of intestinal health. Total RNA from the ileum mucosa was extracted using TRIzol reagent (Vazyme Biotech Co., Ltd.). Total RNA was purified through lithium chloride precipitation. The TaKaRa PrimeScript™ RT reagent Kit (TaKaRa, Beijing, China) was used for cDNA synthesis, and RT-PCR was performed using ChamQ SYBR Color qPCR Master Mix (Vazyme Biotech Co., Ltd.) according to the manufacturer's instructions. Relative mRNA expressions were quantified using the threshold cycle (2^−ΔΔCt^) method, as described previously (Livak and Schmittgen, 2001). The primers used are listed in Table 2.

**Table 2:** Primers used in this study.

Genes	Forward Primer (5′−3′)	Reverse Primer (5′−3′)
*β-actin*	TGCTGTGTTCCCATCTATCG	TTGGTGACAATACCGTGTTCA
*ZO-1*	ACACAGCTCATCACAGCCTC	TCCTCTAGTGCTGAAGGGCT
*occludin*	GTGTAAGGCCCACACCTCTG	TGAGCTGTGTGCTCAGGGTA
*TLR4*	TCATGGCACCTACCCTGTCT	TCTCAAAGGAGTTGCCTGCC
*MUC2*	ACCACCACAACACCCTTCAG	GGTGGAGGAGAGTGGGTTTG
*LYZ*	ATGGGAGTACCGACTACGGA	CACGCTCGCTGTTATGTCTG

MUC2, Mucin 2; TLR4, Toll-like receptor 4; LYZ, lysozyme; ZO-1, Zonula occludens protein 1.

### Transcriptome sequencing

Total RNA from the ileum mucosa was extracted using TRIzol reagent (Vazyme Biotech Co., Ltd.). RNA integrity was assessed using a Bioanalyzer 2100 and confirmed through 1 % agarose gel electrophoresis. High-quality RNA was used to construct cDNA libraries for sequencing. The cDNA was subjected to 200 paired-end sequencing on the Illumina NovaSeq™ 6000 platform in PE150 mode. Raw sequencing data produced by RNA sequencing underwent quality control, which involved the removal of reads with low-quality bases and adapter contamination. Gene expression levels were evaluated as fragments per kilobase per million mapped reads. The R package DESeq2 was used to analyze differentially expressed genes (**DEGs**). Adjusted *P* < 0.05 and |log2 fold change | > 1 were set as the thresholds for DEGs, which were visualized using a volcano plot. Functional enrichment analysis of the DEGs was performed using the R package clusterProfiler, which included Gene Ontology (**GO**) and Kyoto Encyclopedia of Genes and Genomes **(KEGG**) analyses. Gene Set Enrichment Analysis (**GSEA**) was used to analyze the enrichment of the gene dataset between the groups. GSEA relied on the KEGG database and involved calculating a normalized enrichment score for each gene set through 1,000 permutations of the genome. |Normalized enrichment score| > 1 and FDR < 0.05 were set as the cutoff criteria for the GSEA analysis.

### 16S rRNA gene sequencing

Total genomic DNA from the cecal content of chickens was extracted using a QIAamp DNA isolation kit (Qiagen, Hilden, Germany) according to the manufacturer's instructions. The highly variable V3 to V4 region of the 16S rRNA gene was amplified. Sequencing reads were denoised using the DADA2 method in QIIME2 to split the amplicon sequence variants (**ASVs**). The SILVA database (version 138) was used as a reference to classify and annotate each representative sequence of ASVs using a naïve Bayes classifier with a confidence threshold of 70 %. Raw data were analyzed using the QIIME2 platform. Differences in the relative abundances of gut microbiota between groups were compared using linear discriminant analysis effect size (**LEfSe**) with LEfSe software. Species with a linear discriminant analysis value ≥ 2.5 were considered biomarkers. Microbiome function was predicted using PICRUSt2.

### Untargeted metabolomics analysis

Metabolome analysis was performed using an ultra-performance liquid chromatography system (Acquity I-Class PLUS) coupled with a quadrupole time-of-flight high-resolution mass spectrometer (Xevo G2-XS) equipped with an electrospray ionization source. The raw data were processed using Progenesis QI software for peak detection, calibration, and other data-processing operations. For the two-group analysis, differential metabolites were determined by VIP (VIP > 1) and *P*-value (*P* < 0.05, Student's t-test). VIP values were extracted from the OPLS-DA results generated using the R package MetaboAnalystR. Identified metabolites were annotated using the KEGG Compound database, and annotated metabolites were mapped to the KEGG Pathway database. Significantly enriched pathways were identified using a hypergeometric test (*P* < 0.05) for differentially expressed metabolites.

### Statistical analysis

Data are presented as the mean ± standard error of the mean. An unpaired Student's *t*-test was performed to analyze the differences between the two groups using the Prism 8.5 program (GraphPad Software, San Diego, USA). Statistical significance was set at *P* < 0.05.

## Results

### Effect of supplementation with SBP on the growth performance and intestinal immunity of chickens

Growth performance is presented in Table 3. Compared to the control group, the average daily weight gain of the SBP group increased significantly by 4.05 % (*P* < 0.05), and the ratio of feed intake to weight gain decreased significantly by 2.6 % (*P* < 0.05). Intestinal immunity traits are presented in Table 4. Compared with the control group, the SBP group showed a significant reduction in the levels of proinflammatory factors IL-1β and IFN-γ in the ileum mucosa of chickens (*P* < 0.05), while showing a significant increase in the levels of anti-inflammatory factors IL-10 and SIgA (*P* < 0.05). There were no significant differences in the other traits between the two groups (*P* > 0.05).

**Table 3:** Growth performance of broiler chickens.

Index	Control	SBPG	SEM	*P value*
Initial body weight, g	38.61	38.67	0.20	0.772
Final body weight, g	2819	2931	46.33	0.026
Average daily gain, g	40.30	41.93	0.61	0.017
Average daily feed intake, g	97.44	98.78	1.49	0.368
Feed conversion ratio	2.42	2.36	0.02	0.015

In the same row, the *P* < 0.05 indicates statistically significant. SEM, standard error of the mean; SBPG, soybean bioactive peptide experimental group.

**Table 4:** Concentrations of immune factors in the ileum mucosa of broiler chickens.

Items	Control	SBPG	SEM	*P* value
IL-1β, pg/ml	78.09	66.29	3.25	0.003
IL-6, pg/ml	2.58	2.55	0.12	0.844
IFN-γ, pg/ml	18.21	15.85	0.53	0.001
TNF-α, ng/ml	6.45	6.28	0.31	0.603
IL-10, pg/ml	6.25	7.33	0.19	<0.001
SIgA, ng/ml	212	260	12.17	0.002

IFN-γ, interferon-γ; TNF-α, tumor necrosis factor-α; SEM, standard error of the mean; SBPG, soybean bioactive peptide experimental group; SIgA, secretory immunoglobulin A.

### Effect of supplementation with SBP on the intestinal health of chickens

The results of the morphological analyses are presented in Table 5. Compared with the control group, the SBP group showed a significant increase in ileal VH, muscular thickness, and mucosal thickness of the ileal tissue (*P* < 0.05). There were no significant differences in the other traits between the two groups (*P* > 0.05).

**Table 5:** Intestinal morphological indicators of broiler chickens.

Items	Control	SBPG	SEM	*P* value
Villus height, μm	1031	1179	47.10	0.004
Crypt depth, μm	179	198	13.50	0.166
Villus height/Crypt depth	5.96	6.08	0.37	0.743
Muscular thickness, μm	239	338	37.03	0.024
Goblet cell number, cells/mm^2^	2495	2769	249	0.299
Mucosal thickness, μm	17.37	33.32	4.39	0.005

SEM, standard error of the mean; SBPG, soybean bioactive peptide experimental group.

**Fig. 1:**
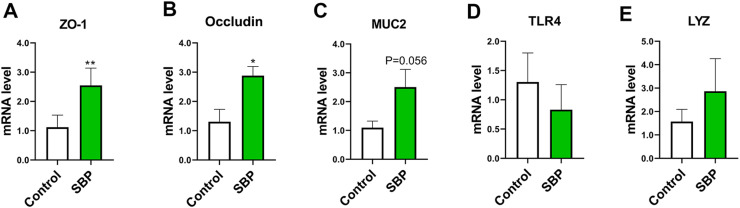
mRNA expression levels of intestinal barrier-related and immune-related genes in the ileum mucosa of broiler chickens. (A) Zonula occludens protein 1 (ZO-1) mRNA level. (B) Occludin mRNA level. (C) Mucin 2 (MUC2) mRNA level. (D) Toll-like receptor 4 (TLR4) mRNA level. (E) Lysozyme (LYZ) mRNA level. * Means *P* < 0.05; ** means *P* < 0.01; *n* = 6. SBP, soybean bioactive peptide.

**Fig. 2:**
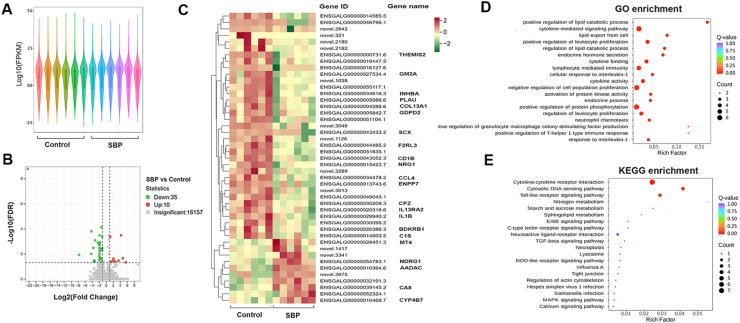
Transcriptome sequencing analysis of ileum mucosa of broiler chickens. (A) Fragments per kilobase per million mapped reads distribution between the control group and SBP group. (B) Volcano plot of differential genes in the gut. (C) Heatmap of 45 significantly differentially expressed genes. (D) GO enrichment analysis of the differentially expressed genes. (E) KEGG enrichment analysis of the differentially expressed genes. SBP, soybean bioactive peptide; GO, Gene Ontology; KEGG, Kyoto Encyclopedia of Genes and Genomes.

The results of quantitative RT-PCR analysis are shown in Fig. 1. Compared to the control group, the mRNA expression levels of zonula occludens protein 1 (*ZO-1)* and *occludin* in the ileum mucosa were significantly upregulated in the SBP group (*P* < 0.05) (Fig. 1A and B). There were no significant differences in the expression levels of mucin 2 protein*,* Toll-like receptor 4, or lysozyme between the two groups (*P* > 0.05) (Fig. 2C–E).

### Supplementing SBP changed genes related to intestinal immune and metabolic processes in chickens

To further understand the mechanisms underlying the changes caused by SBP in the intestine, transcriptome sequencing analysis was performed to explore the overall changes in intestinal genes in the ileal mucosa of the two groups of chickens (Fig. 2). The fragments per kilobase per million mapped reads distribution between the control and SBP group ileal samples was not the same, indicating that the overall gene expression levels were different (Fig. 2A). Compared to the control group, the SBP group had 45 DEGs, including 10 upregulated and 35 downregulated genes (Fig. 2B and C). Several genes involved in inflammation (*CCL4, IL-1B, IL13RA2, MT4*, and so on) were significantly downregulated in the ileum of the SBP group.

GO enrichment analysis was performed to explore the physiological functions of the DEGs (Fig. 2D). The results showed that most of the significantly enriched GO entries included “positive regulation of lipid metabolism process,” “positive regulation of granulocyte macrophage colony-stimulating factor production,” “positive regulation of T-helper 1 type immune response,” “lipid export from cell,” and “regulation of lipid metabolism process.” Furthermore, KEGG metabolism pathway enrichment analysis showed that the DEGs were significantly enriched in the “Cytokine-cytokine receptor interaction,” “Cytosolic DNA-sensing pathway,” and “Toll-like receptor signaling pathway” (Fig. 2E).

**Fig. 3:**
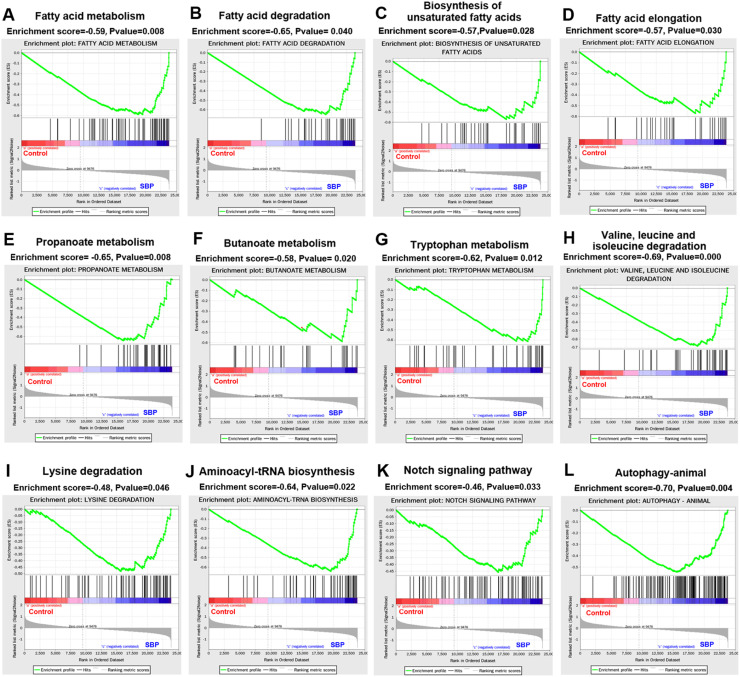
Changes in metabolic pathways caused by SBP revealed by Gene Set Enrichment Analysis (GSEA). (A) Fatty acid metabolism. (B) Fatty acid degradation. (C) Biosynthesis of unsaturated fatty acids. (D) Fatty acid elongation. (E) Propanoate metabolism. (F) Butanoate metabolism. (G) Tryptophan metabolism. (H) Valine, leucine, and isoleucine degradation. (I) Lysine degradation. (J) Aminoacyl-tRNA biosynthesis. (K) Notch signaling pathway. (L) Autophagy-animal. SBP, soybean bioactive peptide.

In addition, GSEA was performed to explore the possible pathways and potential biological mechanisms related to the genes associated with the SBP group (Fig. 3). Fatty acid metabolism, fatty acid degradation, unsaturated fatty acid biosynthesis, fatty acid elongation, propanoate metabolism, butanoate metabolism, tryptophan metabolism, valine, leucine, and isoleucine degradation, lysine degradation, aminoacyl-tRNA biosynthesis, the Notch signaling pathway, and autophagy were significantly enriched in the SBP group (*P* < 0.05). Surprisingly, no pathway was significantly enriched in the control group.

### Effect of supplementation with SBP on the gut microbiota of chickens

**Fig. 4:**
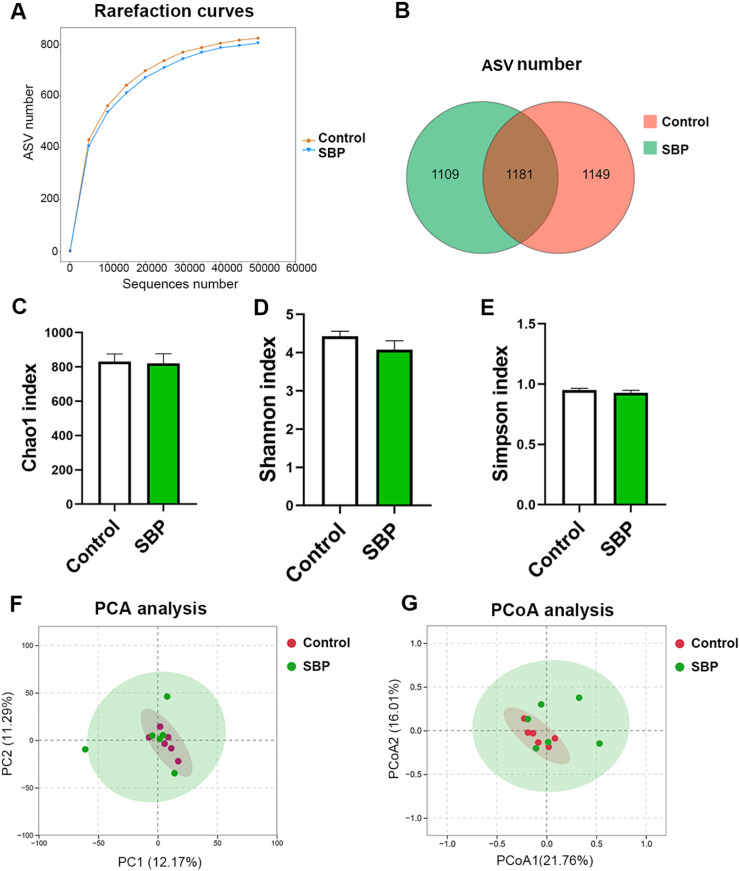
16S rRNA sequencing analysis of the cecal microbiota in chickens. (A) Rarefaction curves of sequencing. (B) Venn diagram of the number of shared and unique ASVs. (C) Chao1 index. (D) Shannon index. (E) Simpson index. (F) Principle component analysis (PCA) analysis of microbiota at the ASV level. (G) Principal coordinate analysis (PCoA) analysis of microbiota at the ASV level. SBP, soybean bioactive peptide; ASV, amplicon sequence variant.

**Fig. 5:**
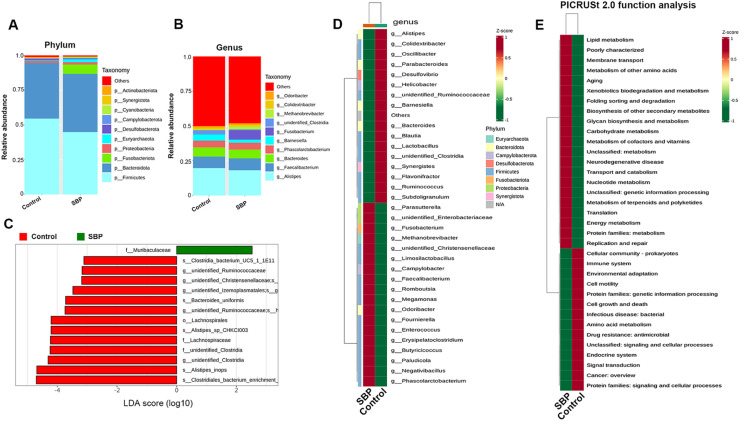
Cecal microbiota structure and PICRUSt function analysis in broilers. (A) Column chart of species composition at the phylum level. (B) Column chart of species composition at the genus level. (C) Histogram of the linear discriminant analysis (LDA) scores computed for differentially abundant bacterial taxa. (D) Heatmap of 35 bacterial genera with high abundance between groups. (E) Differential analysis of functional predictions in two groups. SBP, soybean bioactive peptide.

16S rRNA sequencing was used to analyze the diversity and composition of the cecal microbiota of chickens (Figs. 4 and 5). Fig. 4A shows that the sequencing depth was sufficient to cover the microbial diversity in each group. The Venn diagram in Fig. 4B shows that there were 1,181 common ASVs shared by both groups, accounting for 34.34 %; 1,149 unique ASVs in the control group, accounting for 33.41 %; and 1,109 unique ASVs in the SBP group, accounting for 32.25 %. However, there were no significant differences in the Chao1, Shannon, and Simpson indices between the two groups (*P* > 0.05) (Fig. 4C–E). Principal component analysis and Bray–Curtis principal coordinate analysis were used to determine the beta diversity of bacterial communities (Fig. 4F and G). Both results showed that discrimination between the groups was not significantly different; however, the microbial composition showed a high degree of aggregation within each group.

The composition of chicken cecal microbiota at the phylum level is shown in Fig. 5A. *Firmicutes* (54.58 %), *Bacteroidota* (40.06 %), and *Proteobacteria* (1.33 %) were the top three dominant taxa in the control group, whereas *Firmicutes* (44.94 %), *Bacteroidota* (41.75 %), and *Fusobacteriota* (6.72 %) were the top three dominant taxa in the SBP group. At the genus level (Fig. 5B), more *Fusobacterium* and *Methanobrevibacter* genera were present in the SBP group, whereas the abundances of *Barnesiella* and *Unidentified_Clostridia* genera were significantly reduced. To identify specific individual bacterial taxa in the two groups, we performed LEfSe analysis using a linear discriminant analysis score ≥ 2.5 and *P* < 0.05 as criteria to determine whether there were differences in the biomarkers between the two groups. Thirteen bacterial taxa were significantly enriched in the control group, and only one bacterial taxon (*Muribaculaceae* family) was highly enriched in the SBP group (Fig. 5C). Hierarchical cluster analysis, visualized using a heat map, revealed 35 abundant taxa that were significantly different between the groups at the genus level (Fig. 5D).

To investigate the functional profiles of the gut microbiota composition between the two groups, PICRUSt 2.0 functional prediction was performed using KEGG pathway analysis (Fig. 5E). Similar to the results of the intestinal transcriptome analysis, the functional prediction of the microbiota showed that 21 KEGG metabolic terms (level 2) were significantly enriched in the SBP group, including lipid metabolism, metabolism of other amino acids, glycan biosynthesis and metabolism, carbohydrate metabolism, and metabolism of cofactors and vitamins.

### Effect of supplementation with SBP on the intestinal metabolic profile of broilers

**Fig. 6:**
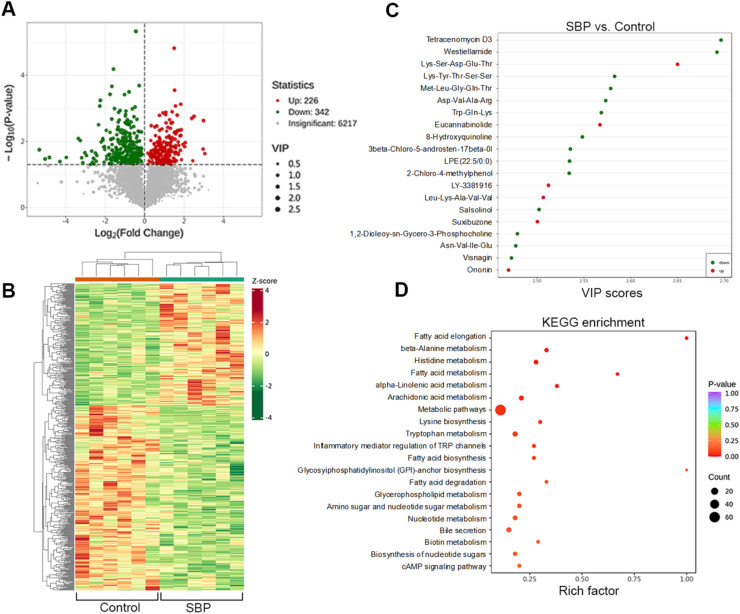
Non-targeted metabolomic analysis of cecal contents. (A) Volcano plot of differential metabolites. (B) Cluster heatmap of all differential metabolites. (C) Top 20 changed metabolites with VIP values. (D) KEGG metabolism enrichment analysis of changed metabolites. SBP, soybean bioactive peptide; Kyoto Encyclopedia of Genes and Genomes.

Subsequently, a non-targeted metabolomic analysis based on HPLC-MS/MS was performed to reveal the details of how SBP supplementation alters the composition of intestinal metabolites in broiler chickens (Fig. 6). The results showed that 568 metabolites differed significantly in the cecal contents between the two groups of chickens. Compared with the control group, 226 metabolites were upregulated, and 342 metabolites were downregulated in the SBP group (Fig. 6A). The cluster heatmap in Fig. 6B shows the total differences in altered metabolites between the two groups of chickens. The top 20 differential metabolites (*P* < 0.05, VIP > 2.0), including six upregulated metabolites and 14 downregulated metabolites, are shown in Fig. 6C, sorted by their VIP scores. Furthermore, KEGG enrichment analysis was applied to clarify the relevant metabolic changes responsible for the formation of differential metabolites. Differential metabolites were mainly enriched in pathways, such as fatty acid biosynthesis, GPI anchor biosynthesis, fatty acid metabolism, and alpha-linolenic acid metabolism (Fig. 6D).

## Discussion

Gut health plays a critical role in the efficiency and overall performance of poultry. Intestinal damage commonly occurs during the growth of yellow-feathered broilers under intensive, antibiotic-free feeding practices. This deterioration in intestinal health leads to reduced productivity among broilers, posing an urgent challenge. Increasing evidence supports the beneficial effects of SBP in alleviating intestinal damage induced by environmental stress, thereby promoting host health and enhancing animal growth performance (Zuo et al., 2013; Osho et al., 2019; Kim et al., 2021; Bai and Zhang, 2023; Sa'adoon and Abbas, 2023). Our data demonstrated that SBP facilitates the rapid growth of broilers by boosting immune function, enhancing ileal morphology, preserving intestinal barrier integrity, modulating intestinal microbiota composition, and optimizing metabolic function.

Gut health can effectively improve animal health and growth performance (Chen et al., 2024). In this study, we found that SBP supplementation significantly increased the concentration of SIgA and decreased proinflammatory factors, such as IL-1β, IFN-γ, and TNF-α, in the ileum mucosa of yellow-feathered broilers. SIgA is a subtype of immunoglobulin A that not only has anti-inflammatory and anti-infection properties but also prevents the adhesion of pathogens, thereby strengthening the intestinal immune barrier and protecting the intestinal mucosal structure (Corthesy, 2013; Monteiro, 2014). Consistent with our current results, several previous reports have indicated that peptides derived from dietary protein sources, including soybean, corn, and egg white, downregulate the expression of IL-1β, IFN-γ, and TNF-α while upregulating the anti-inflammatory mediator IL-10 (Majumder et al., 2016; Carlini et al., 2023).

In addition, our transcriptome results confirmed that SBP positively regulated the immune response in the ileal mucosa of broilers. In general, changes in inflammatory cytokines are modulated through Toll-like receptor pathways (Gao et al., 2020; Al-Sadi et al., 2021). The involvement of this pathway was substantiated in this study, where KEGG enrichment analysis revealed significant enrichment of Toll-like receptor pathways and cytokine-cytokine interactions. Furthermore, we propose that the reduced inflammation in broilers may be linked to improvements in tryptophan metabolism. GSEA showed that tryptophan metabolism improved in the SBP group in the present study. Tryptophan exhibits notable anti-inflammatory effects in vivo by modulating the activities of indoleamine 2,3-dioxygenase, kynurenine, and aryl hydrocarbon receptors (Sorgdrager et al., 2019). Consequently, SBP potentially influences immune responses by regulating the Toll-like receptor pathway and enhancing tryptophan metabolism, thereby reducing inflammation and preserving the intestinal health of broilers.

The higher the VH, the shorter the crypt depth, which is more favorable for intestinal nutrient absorption; otherwise, it leads to poor nutrient absorption and impaired overall performance (Zhang et al., 2020). Notably, we showed that the SBP group of broilers had more uniform intestinal villi and higher VH, indicating a greater nutrient absorption capacity, which is similar to the results of Wei et al. (2024). This partly explains why the feed conversion ratio of the broilers improved. We also hypothesized that the improvement in intestinal morphology may be related to the activation of the Notch signaling pathway, as the Notch signaling pathway can repair damaged morphological structures by promoting the proliferation and differentiation of intestinal cells (VanDussen et al., 2012).

The intestinal mucosal barrier acts as a physical and chemical barrier to the external environment and is essential for protecting the gut (Okumura and Takeda, 2018). In this study, SBP improved intestinal physical and chemical barrier functions. Tight junction proteins (such as ZO-1 and occludin) are important components of physical barriers (Suzuki, 2013). Stress-induced tight junction damage can cause systemic inflammation and adversely affect the growth of broilers (Rostagno, 2020), whereas small peptides can maintain the integrity of tight junctions by upregulating the expression of *ZO-1* and *occludin* in the intestine (Zhao et al., 2022). These findings are in agreement with the current results. In addition, this study showed significant upregulation of *MUC-2* in the SBP group. MUC-2 is a secreted mucin that not only forms a mucus barrier to protect the gut but also exerts anti-inflammatory and antimicrobial activity through its O-glycans (Bergstrom and Xia, 2022).

Dietary peptides have been shown to optimize the composition of the gut microbiota (Mu et al., 2023). We found that SBP supplementation positively affected the intestinal microbiota of broilers. Our LEfSe analysis showed significant enrichment of *Muribaculaceae* in the cecum of broilers in the SBP group. *Muribaculaceae* can improve intestinal barrier integrity and thus play an important role in maintaining intestinal homeostasis (Tang et al., 2024; Zhu et al., 2024). In addition, *Muribaculaceae* has the potential to improve metabolic disorders and promote growth (Yang et al., 2024; Zhu et al., 2024). SBP also increased the abundances of *Fusobacterium* and *Methanobrevibacter. Fusobacterium* can inhibit the growth of *Salmonella enteritidis*, which is beneficial for maintaining intestinal homeostasis and health (Portrait et al., 2000). *Methanobrevibacter* affects host energy metabolism and has been shown to be associated with weight gain in poultry (Ji et al., 2019; Marková et al., 2024).

The intestinal metabolic characteristics of yellow-feathered broilers were significantly altered after SBP intervention; in particular, lipid-related pathways, such as GPI anchor biosynthesis, alpha-linolenic acid metabolism, and other fatty acid metabolic pathways, were significantly enhanced. GPI is a unique glycolipid that plays an important role in intestinal health by maintaining intestinal immune homeostasis and epithelial barrier integrity (Chisholm et al., 2015; Jangid et al., 2022). As a polyunsaturated fatty acid, alpha-linolenic acid not only improves carcass quality by regulating lipid metabolism but also has anti-inflammatory, anti-pathogenic, and antioxidant biological activities (Long et al., 2020; Zhu et al., 2020; Liu et al., 2022). Notably, the abundance of Muribaculaceae is highly correlated with changes in lipid compounds, favoring improved lipid metabolism (Zhang et al., 2021). Therefore, we speculate that SBP may enhance lipid-related metabolic pathways by promoting Muribaculaceae growth, thereby maintaining intestinal homeostasis and improving broiler growth performance.

## Conclusion

The administration of SBP improved the growth performance and health status of yellow-feathered broilers. Regarding the underlying mechanism, the improvement of gut health by SBP is key. These improvements include enhancing intestinal immune homeostasis, promoting intestinal development, maintaining intestinal barrier integrity, optimizing the composition of intestinal microbiota, and optimizing metabolic function.

## Disclosures

The authors declare no conflicts of interest.

## References

[bib0001] Abdollahi M.R., Zaefarian F., Gu Y., Xiao W., Jia J., Ravindran V. (2017). Influence of soybean bioactive peptides on growth performance, nutrient utilisation, digestive tract development and intestinal histology in broilers. J. Appl. Animal Nutr..

[bib0002] Al-Sadi R., Dharmaprakash V., Nighot P., Guo S., Nighot M., Do T., Ma T.Y. (2021). Bifidobacterium bifidum enhances the intestinal epithelial tight junction barrier and protects against intestinal inflammation by targeting the toll-like receptor-2 pathway in an NF-κb-independent manner. Int. J. Mol. Sci..

[bib0003] Bergstrom K., Xia L.J. (2022). The barrier and beyond: roles of intestinal mucus and mucin-type O-glycosylation in resistance and tolerance defense strategies guiding host-microbe symbiosis. Gut. Microb..

[bib0004] Bai B.L., Zhang H.F. (2023). Effect of soybean active peptides on growth performance, carcass traits, serum biochemical indexes and intestinal microorganisms in finishing pigs. Feed Res.

[bib0005] Corthesy B. (2013). Multi-faceted functions of secretory IgA at mucosal surfaces. Front. Immunol..

[bib0006] Chisholm A.D., Budirahardja Y., Doan T.D., Zaidel-Bar R. (2015). Glycosyl phosphatidylinositol anchor biosynthesis is essential for maintaining epithelial integrity during Caenorhabditis elegans embryogenesis. Plos Genet.

[bib0007] Chatterjee C., Gleddie S., Xiao C.W. (2018). Soybean bioactive peptides and their functional properties. Nutrients.

[bib0008] Caekebeke N., Ringenier M., De Meyer F., Ducatelle R., Ongena N., Van Immerseel F., Dewulf J. (2020). A study on risk factors for macroscopic gut abnormalities in intensively reared broiler chickens. Avian Pathol.

[bib0009] Carlini V., Noonan D.M., Abdalalem E., Goletti D., Sansone C., Calabrone L., Albini A. (2023). The multifaceted nature of IL-10: regulation, role in immunological homeostasis and its relevance to cancer, COVID-19 and post-COVID conditions. Front. Immunol.

[bib0010] Chen C., Feng F., Qi M., Chen Q., Tang W., Diao H., Hu Z., Qiu Y., Li Z., Chu Y., Tang Z. (2024). Dietary citrus flavonoids improved growth performance and intestinal microbiota of weaned piglets via immune function mediated by TLR2/NF-κb signaling pathway. J. Agric. Food Chem..

[bib0011] Gao H., Kang N., Hu C., Zhang Z., Xu Q., Liu Y., Yang S. (2020). Ginsenoside Rb1 exerts anti-inflammatory effects in vitro and in vivo by modulating toll-like receptor 4 dimerization and NF-kB/MAPKs signaling pathways. Phytomedicine.

[bib0012] Guo P., Lin S., Lin Q., Wei S., Ye D., Liu J. (2023). The digestive tract histology and geographical distribution of gastrointestinal microbiota in yellow-feather broilers. Poult. Sci..

[bib0013] Ji J., Luo C.L., Zou X., Lv X.H., Xu Y.B., Shu D.M., Qu H. (2019). Association of host genetics with intestinal microbial relevant to body weight in a chicken F2 resource population. Poult. Sci..

[bib0014] Jangid A., Fukuda S., Seki M., Suzuki Y., Taylor T.D., Ohno H., Prakash T. (2022). Gut microbiota alternation under the intestinal epithelium-specific knockout of mouse Piga gene. Sci. Rep-Uk..

[bib0015] Karimzadeh S., Rezaei M., Yansari A.T. (2016). Effects of canola bioactive peptides on performance, digestive enzyme activities, nutrient digestibility, intestinal morphology and gut microflora in broiler chickens. Poult. Sci. J..

[bib0016] Kim I.S., Yang W.S., Kim C.H. (2021). Beneficial effects of soybean-derived bioactive peptides. Int. J. Mol. Sci..

[bib0017] Livak K.J., Schmittgen T.D. (2001). Analysis of relative gene expression data using real-time quantitative PCR and the 2−ΔΔCT method. Methods.

[bib0018] Long S., Liu S., Wu D., Mahfuz S., Piao X. (2020). Effects of dietary fatty acids from different sources on growth performance, meat quality, muscle fatty acid deposition, and antioxidant capacity in broilers. Animals.

[bib0019] Landy N., Kheiri F., Faghani M. (2020). Evaluation of cottonseed bioactive peptides on growth performance, carcase traits, immunity, total antioxidant activity of serum and intestinal morphology in broiler chickens. Ital. J. Anim. Sci..

[bib0020] Liu P., Liu M., Liu X., Xue M., Jiang Q., Lei H. (2022). Effect of α-linolenic acid (ALA) on proliferation of probiotics and its adhesion to colonic epithelial cells. Food Sci. Technol..

[bib0021] Monteiro R.C. (2014). Immunoglobulin A as an anti-inflammatory agent. Clin. Exp. Immunol..

[bib0022] Majumder K., Mine Y., Wu J. (2016). The potential of food protein-derived anti-inflammatory peptides against various chronic inflammatory diseases. J. Sci. Food Agric..

[bib0024] Mu J., Lin Q., Liang Y. (2023). An update on the effects of food-derived active peptides on the intestinal microecology. Crit. Rev. Food Sci. Nutr..

[bib0025] Marková K., Kreisinger J., Vinkler M. (2024). Are there consistent effects of gut microbiota composition on performance, productivity and condition in poultry?. Poult. Sci..

[bib0026] Okumura R., Takeda K. (2018). Maintenance of intestinal homeostasis by mucosal barriers. Inflamm. Regen..

[bib0027] Osho S.O., Xiao W.W., Adeola O. (2019). Response of broiler chickens to dietary soybean bioactive peptide and coccidia challenge. Poult. Sci..

[bib0028] Portrait V., Cottenceau G., Pons A.M. (2000). A Fusobacterium mortiferum strain produces a bacteriocin-like substance(s) inhibiting Salmonella enteritidis. Lett. Appl. Microbiol..

[bib0029] Rostagno M.H. (2020). Effects of heat stress on the gut health of poultry. J. Anim. Sci..

[bib0030] Suzuki T. (2013). Regulation of intestinal epithelial permeability by tight junctions. Cell. Mol. Life Sci..

[bib0031] Sorgdrager F.J., Naudé P.J., Kema I.P., Nollen E.A., Deyn P.P.D (2019). Tryptophan metabolism in inflammaging: from biomarker to therapeutic target. Front. Immunol..

[bib0032] Sugiharto S. (2022). Dietary strategies to alleviate high-stocking-density-induced stress in broiler chickens – a comprehensive review. Arch. Anim. Breed..

[bib0033] Sa'adoon W.H., Abbas R.J. (2023). Physiological, immunological and microbial effects of soybean bioactive peptides and vitamin E supplementing to broiler diet. Acta Sci.

[bib0034] Tang Y.F., Xie W.Y., Wu H.Y., Guo H.X., Wei F.H., Ren W.Z., Gao W., Yuan B. (2024). Huaier polysaccharide alleviates dextran sulphate sodium salt-induced colitis by inhibiting inflammation and oxidative stress, maintaining the intestinal barrier, and modulating gut microbiota. Nutrients.

[bib0035] VanDussen K.L., Carulli A.J., Keeley T.M., Patel S.R., Puthoff B.J., Magness S.T., Tran I.T., Maillard I., Siebel C., Kolterud Å., Grosse A.S. (2012). Notch signaling modulates proliferation and differentiation of intestinal crypt base columnar stem cells. Development..

[bib0036] Wei Y., Zhao X., Xu T., Liu Z., Zuo Y., Zhang M., Zhang Y., Yin H. (2024). Soybean bioactive peptide supplementation affects the intestinal immune antioxidant function, microbial diversity, and reproductive organ development in roosters. Animals.

[bib0037] Yang X., Wang J., Cheng J., Zhang D., Huang K., Zhang Y., Li X., Zhao Y., Zhao L., Xu D., Ma Z. (2024). Relationship between sheep feces scores and gastrointestinal microorganisms and their effects on growth traits and blood indicators. Front. Microbiol..

[bib0038] Zuo W.Y., Hong W.M., Chen G. (2013). Effect of low molecular weight soybean peptide on intestinal immunity in weanling piglets. J. Nanjing Agricult. Univ..

[bib0039] Zhang Y., Chen S., Zong X., Wang C., Shi C., Wang F., Wang Y., Lu Z. (2019). Peptides derived from fermented soybean meal suppresses intestinal inflammation and enhances epithelial barrier function in piglets. Food Agric. Immunol..

[bib0040] Zhu X., Wang B., Zhang X., Chen X., Zhu J., Zou Y., Li J. (2020). Alpha-linolenic acid protects against lipopolysaccharide-induced acute lung injury through anti-inflammatory and anti-oxidative pathways. Microb. Pathog..

[bib0041] Zhang Y., Liu Y., Li J., Xing T., Jiang Y., Zhang L., Gao F. (2020). Dietary corn resistant starch regulates intestinal morphology and barrier functions by activating the Notch signaling pathway of broilers. Asian-Australas J. Anim. Sci..

[bib0042] Zhang Y., Gu Y., Chen Y., Huang Z., Li M., Jiang W., Chen J., Rao W., Luo S., Chen Y., Chen J. (2021). Dingxin Recipe IV attenuates atherosclerosis by regulating lipid metabolism through LXR-α/SREBP1 pathway and modulating the gut microbiota in ApoE-/- mice fed with HFD. J. Ethnopharmacol..

[bib0043] Zhao X., Zhang Y., He W., Wei Y., Han S., Xia L., Tan B., Yu J., Kang H., Ma M., Zhu Q. (2022). Effects of small peptide supplementation on growth performance, intestinal barrier of laying hens during the brooding and growing periods. Front. Immunol..

[bib0044] Zhu H., Zhou X., Shen C., Ao Z., Cao X., Song C., Mehmood M.A., Wu T., Mei J., He M., Ma Y. (2024). Bacillus licheniformis-based intensive fermentation of Tibetan tea improved its bioactive compounds and reinforced the intestinal barrier in mice. Front. Microbiol..

[bib0045] Zhu Y., Chen B., Zhang X., Akbar M.T., Wu T., Zhang Y., Zhi L., Shen Q. (2024). Exploration of the muribaculaceae family in the gut microbiota: diversity, metabolism, and function. Nutrients.

